# Triatomine bugs, their microbiota and *Trypanosoma cruzi*: asymmetric responses of bacteria to an infected blood meal

**DOI:** 10.1186/s13071-016-1926-2

**Published:** 2016-12-09

**Authors:** Sebastián Díaz, Bianca Villavicencio, Nathália Correia, Jane Costa, Karen L. Haag

**Affiliations:** 1 Programa de Pós-Graduação em Genética e Biologia Molecular, Universidade Federal do Rio Grande do Sul, Porto Alegre, Rio Grande do Sul Brazil; 2 Programa de Pós-Graduação em Biologia Celular e Molecular, Universidade Federal do Rio Grande do Sul, Porto Alegre, Rio Grande do Sul Brazil; 3 Laboratório de Biodiversidade Entomológica, Instituto Oswaldo Cruz-Fiocruz, Rio de Janeiro, Rio de Janeiro Brazil; 4 Departamento de Genética, Instituto de Biociências, Universidade Federal do Rio Grande do Sul, Porto Alegre, Rio Grande do Sul Brazil

**Keywords:** Triatomine bugs, Chagas disease, *Trypanosoma cruzi*, *Arsenophonus*, Microbiota, Metabarcoding, 16 rRNA

## Abstract

**Background:**

Triatomine bugs (Hemiptera: Reduviidae) are vectors of the flagellate *Trypanosoma cruzi*, the causative agent of Chagas disease. The study of triatomine gut microbiota has gained relevance in the last years due to its possible role in vector competence and prospective use in control strategies. The objective of this study is to examine changes in the gut microbiota composition of triatomines in response to a *T. cruzi*-infected blood meal and identifying key factors determining those changes.

**Results:**

We sampled colony-reared individuals from six triatomine vectors (*Panstrongylus megistus*, *Rhodnius prolixus*, *Triatoma brasiliensis*, *T. infestans*, *T. juazeirensis* and *T. sherlocki*) comparing experimentally *T. cruzi* strain 0354-challenged and non-challenged insects. The microbiota of gut and gonad tissues was characterized using high throughput sequencing of region V3-V4 of bacterial 16S rRNA gene. The triatomine microbiota had a low intra-individual diversity, and a high inter-individual variation within the same host species. *Arsenophonous* appeared as the dominant triatomine bacterial symbiont in our study (59% of the total 16S coverage), but there were significant differences in the distribution of bacterial genera among vectors. In *Rhodnius prolixus* the dominant symbiont was *Pectobacterium*.

**Conclusions:**

*Trypanosoma cruzi*-challenge significantly affects microbiota composition, with challenged vectors harbouring a significantly more diverse bacterial community, both in the gut and the gonads. Our results show that blood-feeding with *T. cruzi* epimastigotes strongly affects microbiota composition in a species-specific manner. We suggest that triatomine-adapted enterobacteria such as *Arsenophonus* could be used as stable vectors for genetic transformation of triatomine bugs and control of Chagas disease.

**Electronic supplementary material:**

The online version of this article (doi:10.1186/s13071-016-1926-2) contains supplementary material, which is available to authorized users.

## Background

Microorganisms living in the midgut of insect vectors have an important role in modulating vector competence, which is the ability to acquire, maintain and transmit pathogens [1]. Microorganisms may interfere with vector competence either by direct interaction with parasites and competition for resources in the gut, or indirectly by inducing vector anti-parasitic activity and humoral immune defence factors [2–5]. Furthermore, some symbiotic bacteria can be genetically modified to express anti-parasitic molecules or proteins that reduce insect fitness, as shown for *Anopheles* mosquitoes and *Glossina* tsetse flies in which transformed *Pantoea* and *Sodalis* symbionts were introduced, respectively [6, 7].

Triatomine bugs are vectors of the protozoan parasite *T. cruzi*, which causes Chagas disease. About 6 to 7 million people are estimated to be infected worldwide, mostly in Latin America where the disease is endemic [8]. In later years, *T. cruzi* became a public health concern also in the United States and other non-endemic countries, mostly due to human immigration from areas of endemicity [9–11]. It has been suggested that transmission might be controlled through the vector microbiota, since some strains of *Serratia marcescens*, a common symbiont of various triatomine species, have trypanolytic activity on several *T. cruzi* strains [12]. Methods for genetically modifying bacterial symbionts to effectively decrease parasite transmission or the development and fecundity of triatomine bugs have been established [13, 14].

The efficacy of such methods in the real world relies on the identification of key factors that influence the establishment of a successful *T. cruzi* infection on one hand, as well as the bacterial colonization of the insect gut on the other hand. A successful trypanosomatid infection can reduce or alter the triatomine gut microbiota composition [15–17]. Moreover, the ability of some *T. cruzi* strains to develop in certain vector species depends on the intrinsic qualities of either the parasite or the insect vector, as well as the resident host gut microbiota [18]. Previous studies using non-culturing based methods [19, 20] identified some triatomine gut microbiota characteristics: first, its diversity within each host is low with only one or few genera being dominant; secondly, some bacterial genera appear to be specific to certain triatomine hosts, i.e. *Rhodococcus* to *Rhodnius* and *Arsenophonus* to *Triatoma*; finally, lab-reared insects lose part of the original microbiota diversity but conserve most of the bacterial groups found in their wild counterparts.

Differently from other hemipterans, triatomine bugs do not possess specialized cells to harbour symbiotic bacteria (bacteriocytes). Most of the microbiota members have an extracellular lifestyle in the midgut lumen, being hypothetically acquired through the consumption of faeces of conspecifics (coprophagy) or by cannibalism [21]. Therefore, defining the key factors that influence triatomine gut microbiota composition remains a challenge, considering the numerous variables that need to be taken into consideration, such as the diversity of host species, symbiont mode of transmission and lifestyle, as well as the influence of *T. cruzi* infection, among other factors.

To evaluate the role of *T. cruzi* on the diversity and composition of triatomine microbiota, we metabarcoded the bacterial 16S rRNA gene of guts and gonads of colony-reared individuals belonging to six triatomine species, and compared insects that received a blood meal containing *T. cruzi* epimastigotes (*T. cruzi*-challenged) with insects that were fed with parasite-free blood (non-challenged).

## Methods

### Insect sampling and dissection

The six species of triatomine bugs analyzed in our study belong to three different genera, i.e., *Panstrongylus megistus*, *Rhodnius prolixus*, *Triatoma infestans*, *T. brasiliensis*, *T. juazeirensis* and *T. sherlocki*, with the last three species corresponding to the so called “*T. brasiliensis* complex” according to Costa et al. [22]. All insects from the same species were adults that came from a single colony. They have been maintained at the Laboratório de Doenças Parasitárias of Oswaldo Cruz Institute (Fiocruz, IOC) in Rio de Janeiro, and date from 1987 to 2011 depending on the species (Additional file 1: Table S1). A control group (unchallenged) was fed with 10 ml citrated rabbit blood (0.1 ml sodium citrate/1 ml blood). A second group (*T. cruzi*-challenged) was fed with 10 ml of rabbit decomplemented blood containing *T. cruzi* epimastigotes. Decomplemented blood was obtained by an initial centrifugation at 3,500 rpm for 10 min to separate the plasma from the erythrocytes. The plasma was discarded and red cells were washed in 3× phosphate-buffered saline (PBS), then resuspended in 10 ml liver infusion tryptose (LIT) medium containing 1.5 × 10^7^ epimastigotes in exponential growth phase per millilitre. We used the *T. cruzi* strain 0354, belonging to the Discrete Typing Unit I (DTU I), isolated from *T. brasiliensis* insects naturally infected from the municipality of Caicó (RN, Brazil). In addition, the gut microbiota of one adult *P. megistus* female collected in a peridomestic area in the municipality of Parobé (RS, Brazil), and positive for *T. cruzi* as confirmed by microscopy, was sampled as well. Dissections were performed for *T. cruzi*-challenged insects at day 10 after the challenge meal. Control and challenged insects were fed and dissected in parallel. Sterile ultrafine scissors and forceps were used to open the ventral side of specimens from the last thoracic segment to the last abdominal segment, and dissect the midgut (stomach and intestine) and gonads. The organs were separated in individual sterile tubes and washed twice with 1 ml 1× PBS before DNA extraction. All procedures were performed under laboratory aseptic conditions.

### DNA extraction and sequencing

DNA from gut and gonads was extracted using DNeasy Blood & Tissue Kit (Qiagen, Hilden, Germany), according to the manufacturer’s protocol, with a previous lysozyme treatment to break the cell walls of gram-positive bacteria. DNA concentrations were determined on a NanoDrop ND-1000 spectrophotometer (Thermo Fisher Scientific Inc., Waltham, Massachusetts, USA). The quality of bacterial DNA was verified using an initial amplification of the 16S rRNA gene, with primers Bakt_341F (5'-CCT ACG GGN GGC WGC AG-3') and Bakt_805R (5'-GAC TAC HVG GGT ATC TAA TCC-3') [23], which generate a fragment of 464 bp corresponding to the V3-V4 regions of the *E. coli* 16S rRNA gene. In addition, detection of *T. cruzi* was performed by PCR of a 180 bp fragment of the parasite satellite DNA using primers TcZ1 and TcZ2 [24]. For 16S rDNA amplicon sequencing, PCR was performed in triplicates, using modified versions of the original primers, in which an individual tag barcode of 8 nt was added to the 5' end (Additional file 1: Table S1). PCR reactions were performed in a total volume of 25 μl containing 2 μl of total DNA (200 ng on average), 1× PCR buffer, 1.5 mM MgCl_2_, 0.2 mM of each dNTP, 1.25 U of GoTaq DNA polymerase (Promega, Madison, WI, USA), and 0.4 mM of each primer. DNA contamination was controlled by performing negative PCR reactions with 2 μl of sterile water or with 2 μl of the eluate of a DNeasy column that went through the kit protocol but no tissue was added. PCR included an initial denaturation at 95 °C for 5 min, followed by 20 cycles of 94 °C denaturation for 30 s, 55 °C annealing for 30 s (with a touchdown of 0.5 °C every cycle) and 72 °C extension for 20 s, and another 15 cycles with an annealing temperature of 45 °C, with a final extension step at 72 °C for 10 min. PCR products were visualized by electrophoresis on 1.5% agarose gels, and the remaining amplicon volume was purified using PureLink PCR Purification Kit (Invitrogen, Carlsbad, CA, USA). The final products were pooled and 2 × 300 paired-end sequenced on an Illumina Miseq flowcell using the Illumina MiSeq Reagent Kit Version3 (Illumina, San Diego, CA, USA) at Fasteris facilities (Geneva, Switzerland).

### Sequence processing and bacteria identification

Sequencing reads were processed using Mothur v. 1.36.1 [25]. Forward and reverse paired end reads were merged and assigned to their respective replicate according to the tag barcodes. Sequences shorter than 250 bp, containing ambiguous bases, with homopolymer stretches longer than 15 bases or having mismatches in primer sequences, were discarded. Non-bacterial sequences were removed by performing a preliminary classification using the SILVA v119 nr database [26]. Chimeric sequences were removed with Mothur’s implementation of UCHIME [27]. A final dataset was obtained in which singleton sequences as well as samples with a number of sequences equal or smaller than the highest number of sequences obtained for negative controls (2,000 sequences) were discarded. Finally, Operational Taxonomic Units (OTUs) were identified at 97% sequence similarity using the nearest neighbour option, and classified with a confidence threshold of 80% with the SILVA database ver. 119 [26]. Phylogenetic inferences were made to verify the relationships of our most abundant OTUs with other known bacteria. We searched for the most similar 16S sequences to each OTU in the GenBank non-redundant database. The multiple alignment was made with MAFTT v. 7.187 [28], and phylogenetic analyses were performed with PhyML [29] as implemented in Geneious ver 8.1.5 (Biomatters, Auckland, New Zealand) using the GTR + G + I model. Local support values were estimated by nonparametric bootstrap based on 100 re-samplings.

### Alpha and beta diversity analyses

Rarefaction curves and alpha-diversity estimators, i.e. sample coverage (Good’s coverage), richness (Chao1 index), and diversity (Shannon and Inverse Simpson index), were obtained with Mothur for each individual host. Mann-Whitney U-tests were used to evaluate the chance that a random sampling would result in differences between alpha-diversity means as far apart as those observed in our samples. To analyse the influence of host species, host gender, tissue and *T. cruzi*-challenge on bacterial community composition, we carried out a two-way Permutational Multivariate Analysis of Variance (PERMANOVA) of Bray-Curtis dissimilarities with Past 3.13 [30], in which these factors were tested as sources of variation in bacterial community composition. A Redundancy Analysis (RDA) was performed to discriminate the relative degree of influence of each of the above variables on microbiota composition using the *vegan* package [31] in R. As strongly skewed OTU distributions can bias the ordinations, we evaluated different transformation approaches in a Detrended Canonical Correspondence Analysis (DCA) to find one that meets the linearity assumption of RDA. Accordingly, we found that the Hellinger transformation was the best model of community composition, with the longest axis being equal to 2.28. To qualitatively identify the environmental sources of variation that most significantly contribute to the variation in bacterial community composition, a Monte Carlo permutation test and a variance partitioning analysis were used based on the RDA output.

## Results

### Characterization of triatomine bacterial communities

**Fig. 1:**
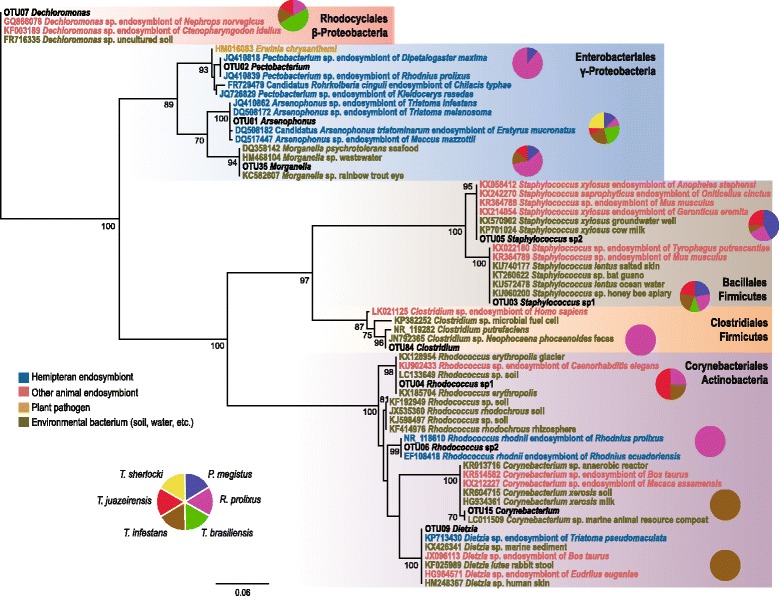
Phylogenetic relationships of the 11 representative OTUs. The phylogeny includes the most closely related sequences of each OTU indicated by their GenBank accession numbers, and shown in different colours according to their origin. Pie charts display the frequency of samples (by host species) in which each OTU was found with at least 10% of the total coverage. Numbers at the internal nodes represent bootstrap support values (≥ 70%)

**Fig. 2:**
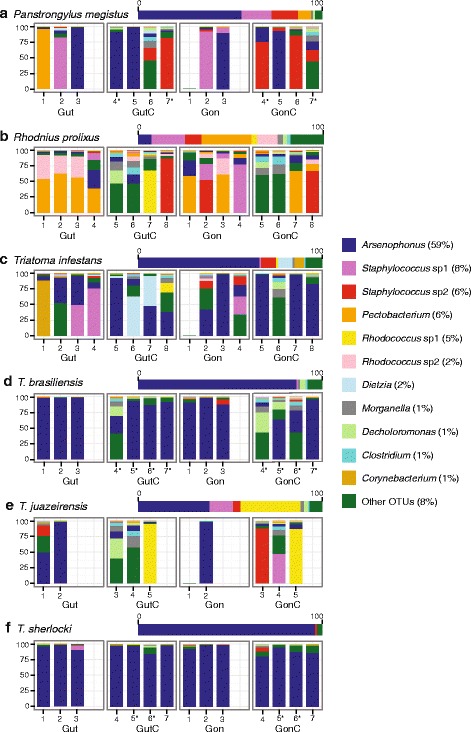
Relative abundance of OTUs in the six species of triatomine bugs (**a-f**). Horizontal bars represent frequency estimates per species, and vertical bars, per sample. *Abbreviations*: Gut, gut samples of non-challenged insects; GutC, gut samples from *T. cruzi-*challenged insects; Gon, gonad samples of non-challenged insects; GonC, gonad samples from *T. cruzi*-challenged insects. Numbers identify unique individuals (see Additional file 2: Table S2 for details). Asterisks indicate individuals that showed positive *T. cruzi* infection at day 10 post-challenge

Our work includes a dataset with 81 samples (46 *T. cruzi*-challenged and 35 non-challenged) plus one *T. cruzi*-infected field-collected *P. megistus* female that was used for comparison (Additional file 2: Table S2). Eight insects showed positive *T. cruzi* PCR at day 10 post-challenge, i.e. 4 *T. braziliensis*, 2 *T. sherlocki* and 2 *P. megistus* (Additional file 2: Table S2; Fig. 2). The parasite was not detected in any tissue of *T. cruzi*-challenged *R. prolixus, T. infestans* or *T. juazeirensis* by diagnostic PCR. The 16S rDNA amplicons of lab-reared insects yielded 1,534,192 sequences (mean coverage ± SD, 18,940 ± 11,207 per sample) that were binned into 824 OTUs (mean ± SD, 85 ± 37 per sample; Additional file 3: Table S3). Good’s coverage estimators are close to 1 (Additional file 2: Table S2), i.e. individual samples reflect very well the entire sampled population, and rarefaction curves, where some samples do not reach saturation, indicate an overestimation of low coverage OTUs (Additional file 4: Figure S1). To analyse the distribution of bacterial taxa in our samples we focused on the 11 OTUs belonging to five bacterial orders (GenBank accession numbers KX011883–KX011909) that reached at least 1% of the total coverage, and pool the remaining OTUs in a single group (Figs. 1, 2).

*Arsenophonus* is by far the predominant bacterium in our samples of lab-reared insects, amounting 59% of the total 16S rDNA coverage of our study, and a surprising abundance of two *Staphylococcus* OTUs is found in some samples of different species (Figs. 1, 2). Bacterial community composition in the parasite-free gut is defined by host taxa, i.e. in *R. prolixus* the dominant OTU is *Pectobacterium* while for the *T. brasiliensis* complex, *Arsenophonus* is dominant (Fig. 2). Direct comparison between the gut and gonads of the same host individual suggests that some groups have the capacity to move across tissues within the body cavity, with *Arsenophonus* showing a consistent presence both in gut and gonad samples. These observations are confirmed by PERMANOVA, which showed that the bacterial community composition of different vectors is significantly dissimilar, whereas for distinct tissues no significant dissimilarities are observed (see below).

### Phylogenetic and ecological relationships

Some OTUs identified in our study such as *Staphylococcus*, *Morganella* and *Clostridium*, are found in diverse environments (Fig. 1), linking their presence in our samples to the colony room environment. Four genera were previously described as members of the triatomine gut microbiota, i.e. *Arsenophonus*, *Pectobacterium, Rhodococcus* and *Dietzia* (Fig. 1). The triatomine gut microbiota seems to be dominated by Enterobacteria. *Arsenophonus*, the predominant bacterium of our study, is mainly an insect-associated genus, including common triatomine endosymbionts (Fig. 1). *Pectobacterium*, another Enterobacteriales, was highly abundant *R. prolixus*, where *Arsenophonus* was less dominant (Fig. 2b). Similarly to *Arsenophonus*, the *Pectobacterium* OTU is closely related with other sequences isolated from triatomine bugs, i.e. *R. prolixus* and *Dipetalogaster maximus* (Fig. 1). For *Rhodococcus*, a Corynebacteriales, we identified two OTUs, i.e. *Rhodococcus* sp. 1, which was found in *T. cruzi*-challenged *R. prolixus* and *Triatoma* spp. and *Rhodococcus* sp. 2, found mostly in non-challenged *R. prolixus.* These OTUs fall in separate clusters of our phylogeny (Fig. 1), with *Rhodococcus* sp. 1 being related to soil free-living bacteria, and *Rhodococcus* sp. 2 clustering with other *Rhodnius* endosymbionts. The latter is yet the only known *Rhodococcus* clade associated with insects. *Corynebacterium* and *Dietzia*, other Corynebacteriales, appeared in low frequency in our samples (1 and 2% of the total 16S coverage, respectively, see Fig. 2).

### Factors influencing the structure of bacterial communities

**Fig. 3:**
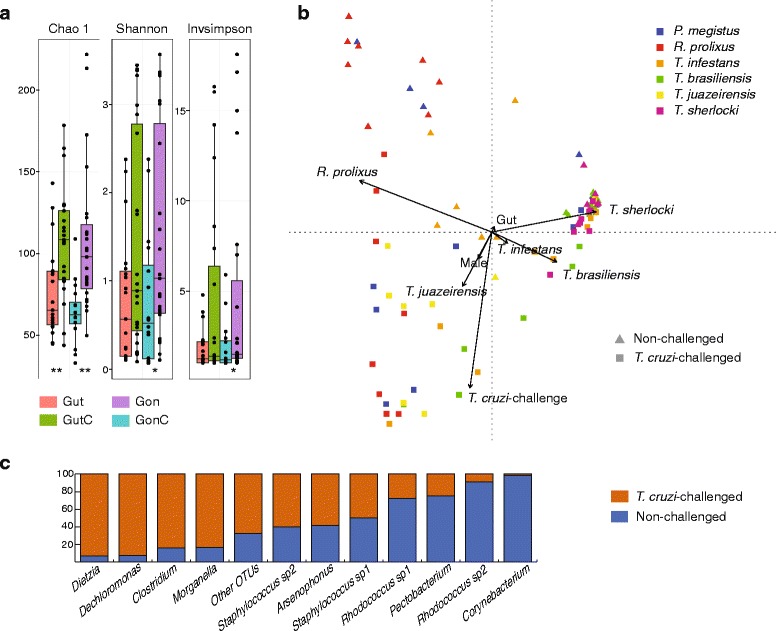
Triatomine alpha- and beta-diversity. **a** Dots show alpha-diversity estimators per sample with their overall distribution displayed as a box-plot. *Abbreviations*: GutC, gut samples from *T. cruzi-*challenged insects; Gon, gonad samples of non-challenged insects; GonC, gonad samples from *T. cruzi*-challenged insects. Asterisks indicate the significant *P*-values of Mann-Whitney U-tests conducted between paired samples (*T. cruzi*-challenged × non-challenged); *P < 0.05, **P < 0.01. **b** Redundancy analysis of bacterial community structure in triatomine bug samples. Independent variables are host species, insect gender, tissue and *T. cruzi*-challenge. Arrow length indicates the strength of correlation between variables and ordination scores. **c** Distribution of OTUs in *T. cruzi*-challenged and non-challenged samples. Bars show the proportion of 16S sequences per OTU found in each group

*Trypanosoma cruzi*-challenged insects bear more diverse bacterial communities (Fig. 3a). The bacterial community of the *T. cruzi*-infected field-collected *P. megistus* female showed the highest alpha-diversity estimates, i.e. Chao1 index = 253.71, Shannon index = 4.22, and Inverse Simpson index = 25.56 (Additional file 2: Table S2); *Arsenophonus* was the most abundant OTU (13.2%; data not shown). To control for variations in their natural habitat, we studied the influence of *T. cruzi*-challenge on the microbiota of laboratory-reared vectors. Though not all experimentally challenged individuals established the infection after 10 days, *T. cruzi*-challenge seems to influence microbiota composition in a species-specific manner. A two-way PERMANOVA, in which host species and *T. cruzi*-challenge were defined as sources of variation of bacterial community composition suggests that both factors, as well as their interaction, contribute to the observed microbiota dissimilarities (Table 1). No significant contributions were detected for tissue or host gender.

**Table 1:** Two-way PERMANOVA of Bray-Curtis dissimilarities in triatomine microbiota composition

Source of variation	Sum of squares	*df*	Mean square	*F*	*P*
*T. cruzi* challenge	0.4638	1	0.4638	2.1811	0.0353
Host species	5.6166	5	1.1233	5.282	0.0001
Interaction	0.2717	5	0.0543	0.2555	0.0003
Residual	14.674	69	0.2126		
Total	21.026	80			
Host gender	0.1024	1	0.1024	0.4201	0.7622
Host species	5.6166	5	1.1233	4.6058	0.0001
Interaction	-1.5215	5	-0.3043	-1.2477	0.1937
Residual	16.829	69	0.2439		
Total	21.026	80			
Tissue	0.0732	1	0.0732	0.2968	0.8935
Host species	5.6166	5	1.1233	4.5532	0.0001
Interaction	-1.6864	5	-0.3372	-1.3671	0.8263
Residual	17.023	69	0.2467		
Total	21.026	80			

*Abbreviation*: *df* degrees of freedom

To evaluate the relative impact of these factors in shaping microbiota structure in the triatomine bugs studied, an ordination analysis (RDA) was performed (Fig. 3b). The analysis showed that all four variables together explain 33.9% of total variance in the community composition (constrained variance). Monte Carlo permutation test (*n* = 199) confirmed that host species (*F* = 5.61, *P* = 0.005) and *T. cruzi*-challenge (*F* = 7.07, *P* = 0.005) are the most significant variables. A variance partitioning of the four factors showed that host species and *T. cruzi*-challenge explain 25.9 and 6.7% of the total variance, respectively, while tissue (gut or gonad) and vector gender have a minimal influence, explaining less than 1% of the variance each.

## Discussion

### Triatomine bacterial symbionts

Under laboratory conditions, the triatomine gut microbiota is characterized by low diversity, being dominated by one or few bacterial genera. Some differences in the taxonomic composition of triatomine gut bacterial communities were found in our study, in comparison with previous results obtained with non-culture- [19, 20] and culture-based methods (reviewed in great extension by Vallejo et al. [32]). A significant absence in our samples is *Serratia*, a genus of bacteria that have trypanolytic activity on specific *T. cruzi* strains [12]. We discarded PCR bias in our experiments as an explanation for such discrepancy, because our primers perfectly match their respective annealing sites in all seven copies of the 16S gene of the *S. marcescens* genome (Additional file 5: Figure S2). Furthermore, OTU160, one of the low frequency OTUs, with coverage of 25×, was identified as *Serratia* (Additional file 3: Table S3). About half (12/25) of the *Serratia* sequences came from the field-collected *P. megistus,* suggesting that its association with the host might be dependent on environmental transmission in the natural habitat. Indeed, its loss under laboratory conditions has been previously described [20].

Interactions with intracellular symbionts that are vertically transmitted (P-symbionts [33]) should be more stable and therefore conserved across individuals of the same host species [34]. In our study, only the presence of *Arsenophonus* was consistent across all six triatomine species, but with varying degrees of dominance. All other bacteria identified in the microbiota of triatomine bugs in this study are clearly facultative (S-symbionts [33]). Most triatomine symbionts are probably extracellular and horizontally transmitted, being able to survive freely outside the host; environmental conditions such as pH, oxygen levels and nutrient availability may act as filters allowing or not their colonization of the gut. Extracellular symbionts of hemipterans are horizontally transmitted by a multitude of routes, with coprophagy, cannibalism and environmental determination being the most prominent transmission strategies (see [34] for a review). The first studies on triatomine gut microbiota transmission hypothesized a horizontal route for the symbiont *Rhodoccocus* in *R. prolixus* via coprophagy of dry excreta [35].

A major exception seems to be *Arsenophonus* [36], which was highly abundant in the gonads of *Triatoma* spp. and *P. megistus* (Fig. 2), concordant with previous histological evidence [37]*.* Unlike other insects, where eggs become infected in early embryonic stages, *Arsenophonus* transmission in *T. infestans* occurs at some later stage, but nevertheless, vertical trans-ovarial transmission is confirmed by the presence of bacteria in the gut of the embryo prior to egg hatching [37]*. Arsenophonus triatominarum*, described by Hypša & Dale [38] has now been identified in 17 species of triatomine bugs and represents the most numerous set of *Arsenophonous* lineages from closely related hosts [39]. Interestingly, aposymbiotic bugs derived by antibiotic treatment remain viable and capable of reproduction, and patterns of molecular evolution of *A. triatominarum*, which include genome degeneration, are thought to be typical of S-symbionts [40].

*Pectobacterium* (named in [19] as "*Candidatus* Rohrkolberia cinguli") was previously found in *R. prolixus, P. megistus* and *Dipetalogaster maxima*. The triatomine *Pectobacterium* symbiont is related to intracellular symbionts of *Cimex lectularius* (bedbug) [41] and *Kleidocerys resedae* (lygaeoid bugs) [42, 43]. In these groups, the bacterium is trans-ovarially transmitted and hosted in a bacteriome. Triatomine bugs do not develop specialized structures to harbor symbiotic bacteria; therefore, further studies are required to determine the habits and modes of transmission of *Pectobacterium* in the Triatominae. However, it is noteworthy that, since *Arsenophonus* and *Pectobacterium* are two core enterobacteria, they may both have similar habits and exploit the same transmission routes.

A second major bacterial order in the triatomine gut microbiota, the Corynebacteriales (*Rhodococcus*, *Dietzia* and *Corynebacterium*), includes extracellular facultative symbionts, common in diverse environments, notably in the soil ecosystem [44]. *Rhodococcus* was shown to be transmitted by coprophagy in *Rhodnius* spp. [35], suggesting that its presence in triatomine hosts should be more dependent on the environment, and maybe even random, in comparison to intracellular taxa. However, extracellular symbionts may exhibit similar mutual patterns of metabolic integration and even co-evolution with their hosts, as much as strictly intracellular symbionts [34], which could be the case of *Rhodococcus*-*Rhodnius* relationship.

### Microbiota interaction with *Trypanosoma cruzi*

Over 60 of the approximate 148 species of triatomine bugs have been found naturally infected or have been experimentally infected with *T. cruzi*, suggesting that probably all species are potential vectors of the parasite [45, 46]. However, different experimental combinations of triatomine species and/or parasite strains show that susceptibility is variable [47], with a tendency of local vectors to be more susceptible to parasite strains of *T. cruzi* from the same geographical areas [48]. In our experimental infection setting, we used a *T. cruzi* strain isolated from the parental population of our *T. brasiliensis* samples. It is therefore not surprising to find that most of the *T. cruzi*-positive samples, as detected by the diagnostic PCR, belong to this species. Two samples of closely related *T. sherlocki*, and the same number of *P. megistus*, were *T. cruzi*-positive as well.

**Fig. 4:**
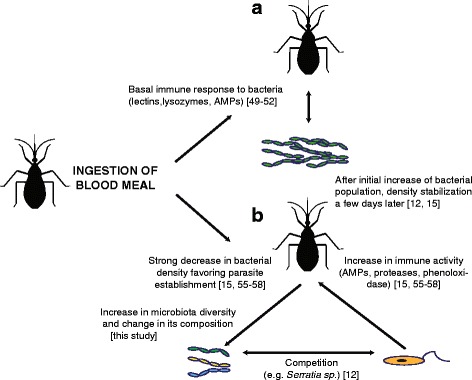
Model of triatomine host-microbiota-parasite interactions based on parasite immune modulation. **a** Triatomine homeostatic responses to blood meal ingestion that control the density of bacteria in the gut. **b** Immune responses induced by *T. cruzi* infection control parasite development, and decrease bacteria density; intense competition for nutrients between bacteria and the protozoan occurs in the gut. An overall increase in micobiota diversity concomitant to infection is observed

We identified *T. cruzi*-challenge as the second most important factor shaping microbiota composition after host species. Such a pattern can be interpreted in the light of a tripartite vector-parasite-microbiota interaction (Fig. 4). After blood ingestion gut bacterial populations increase dramatically, probably due to iron and protein richness of the blood meal [12, 15], which in turn stimulates basal levels of insect immune activity (Fig. 4a). Key triatomine antimicrobial immunity includes recognition molecules such as lectins, lysozymes and antimicrobial peptides (AMPs, e.g. prolixicin and defensins, involved in the Imd immunological pathway) [49–54], responses that decrease bacterial load at approximately one week after feeding [15].

In our study, the microbiota was sampled 10 days post-epimastigote-artificial feeding, a period in which, in natural infection, trypomastigotes from ingested blood transform into replicative epimastigotes in the insect stomach, and in subsequent weeks they migrate to intestine and rectum [18, 55]. This temporal window is not only important for parasite survival, but also for insect homeostasis. For establishing a successful infection, *T. cruzi* modulates vector immunity increasing the basal response against microbial proliferation after feeding, via an intensified activity of defensive routes including AMPs, phenoloxidases and antimicrobial proteases [15, 56–58] (Fig. 4b). In addition, some molecules that are found in the triatomine intestine such as agglutinant lectins and the pigment prodigiosin produced by the symbiont *Serratia* sp. can lyse some *T. cruzi* strains [53, 54]. The intense competition between the parasite and the bacterial community, in addition to homeostatic microbiota-induced immune responses, leads to the ultimate increase in microbiota diversity (Figs. 3a, 4b), probably through the loss of some facultative symbionts and the invasion of more opportunistic bacteria (other OTUs in our study, see Fig. 2).

It is now known that *T. cruzi* reduces vector fitness [59, 60]. In situations where the host is unable to eliminate the parasite via direct immune responses, it may have to rely on its microbiota to fight against the parasite. Indeed, it was shown for the bumblebee-microbiota-trypanosomatid model system, that the microbiota drives both the general host defence against parasites and its specific interaction with different parasite genotypes [61]. In our study, the diagnostic PCR indicates that *T. brasiliensis*, *T. sherlocki* and *P. megistus* are susceptible to infection with *T. cruzi* strain 0354, and we have found *Arsenophonus* to be a dominant bacterium in the same three species. Contrary to *Dietzia*, *Pectobacterium* and *Rhodococcus* that are recognized members of the triatomine microbiota, but were found in our study either mostly in *T. cruzi*-challengend or unchallenged hosts, the prevalence of *Arsenophonus* does not seem to be affected by the parasite (Fig. 3c).

Exploring the tripartite host-microbiota-parasite model, one could predict that a mutualistic symbiotic relationship between bacteria and hosts would evolve in circumstances where the host benefits from decreasing the chance of being infected by the parasite, but parasites in turn should also evolve strategies to evade the defence conferred by symbiotic bacteria. *Arsenophonus* does not seem to be required for triatomine survival or reproduction [40] but it may have been allowed to live inside host cells and possibly being vertically transmitted due to some other benefit conferred to the host, e.g. defence against parasites or nutritional supplement.

## Conclusions

Our study shows that there is a significant increase in diversity, and a change in composition, of the triatomine gut microbiota following the uptake of a *T. cruzi*-infected blood meal. Among all bacteria identified by 16S rRNA gene metabarcoding in our study, *Arsenophonus* seems to be least susceptible to alterations caused by immune responses triggered by feeding, or by the presence of *T. cruzi*, possibly due to its intracellular lifestyle. In the last decade, different suggestions have been made regarding the use of gut microbiota for inhibiting the development of *T. cruzi* in triatomine bugs. We consider that the immune mechanisms used by the insect to control bacterial gut populations, the parasite response to these mechanisms and the functional role of symbionts, are critical to determine the effectiveness of these methodologies. Therefore, we suggest that *Arsenophonus*, with its intracellular lifestyle, is a good candidate for a stable paratransgenesis vector.

## Additional files

Additional file 1: Table S1. Barcodes added to the 16S forward (F) and reverse (R) primers, identifying each amplicon sample. (XLSX 18 kb)
Additional file 2: Table S2. Triatomine sample information. (XLSX 19 kb)
Additional file 3: Table S3. Bacterial OTU identification and coverage. (XLSX 43 kb)
Additional file 4: Figure S1. Rarefaction curves of samples per host species. (PDF 555 kb)
Additional file 5: Figure S2. Metabarcoding primers and their annealing sites on the 7 copies of the 16S rRNA gene of the *Serratia marcescens* genome (avalilable at http://www.ncbi.nlm.nih.gov/genome/1112). (PDF 929 kb)

## Abbreviations

AMP: Antimicrobial Peptide
Imd: Immune deficiency pathway
LIT: Liver Infusion Tryptose
