# Supplementary material for: Triatomine bugs, their microbiota and Trypanosoma cruzi: asymmetric responses of bacteria to an infected blood meal
Source: Parasit Vectors. 2016 Dec 9;9:636. doi: 10.1186/s13071-016-1926-2 (PMC5148865; doi:10.1186/s13071-016-1926-2)
Supplement: Additional file 4: Figure S1. — Rarefaction curves of samples per host species. (PDF 555 kb) [file 13071_2016_1926_MOESM4_ESM.pdf]

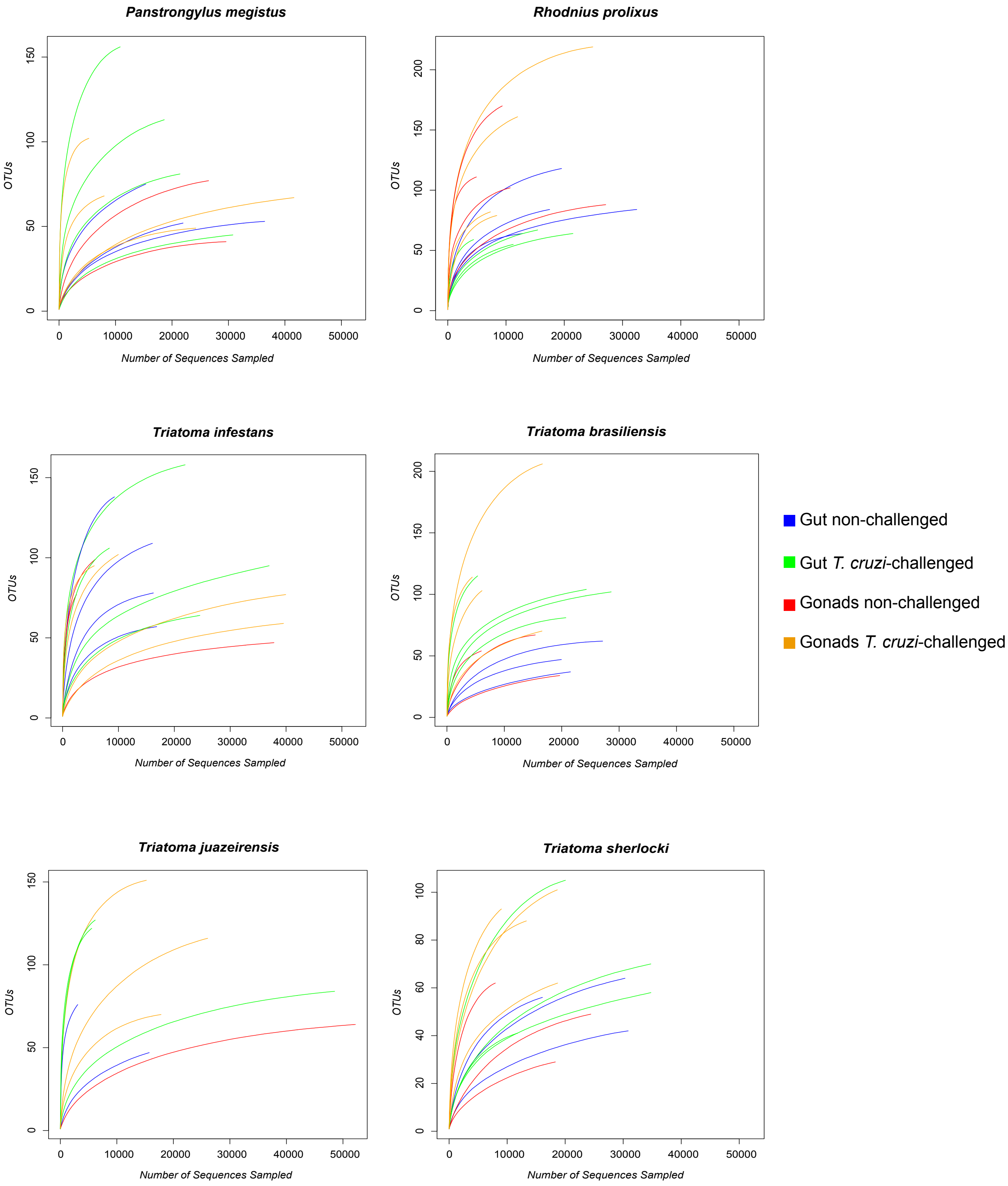

Panstrongylus megistus
Rhodnius prolixus
Triatoma infestans
Triatoma brasiliensis
Triatoma juazeirensis
Triatoma sherlocki
OTUs
Number of Sequences Sampled
0
10000
20000
30000
40000
50000
Gut non-challenged
Gut T. cruzi-challenged
Gonads non-challenged
Gonads T. cruzi-challenged
