# Supplementary material for: Triatomine bugs, their microbiota and Trypanosoma cruzi: asymmetric responses of bacteria to an infected blood meal
Source: Parasit Vectors. 2016 Dec 9;9:636. doi: 10.1186/s13071-016-1926-2 (PMC5148865; doi:10.1186/s13071-016-1926-2)
Supplement: Additional file 5: Figure S2. — Metabarcoding primers and their annealing sites on the 7 copies of the 16S rRNA gene of the Serratia marcescens genome (avalilable at http://www.ncbi.nlm.nih.gov/genome/1112). (PDF 929 kb) [file 13071_2016_1926_MOESM5_ESM.pdf]

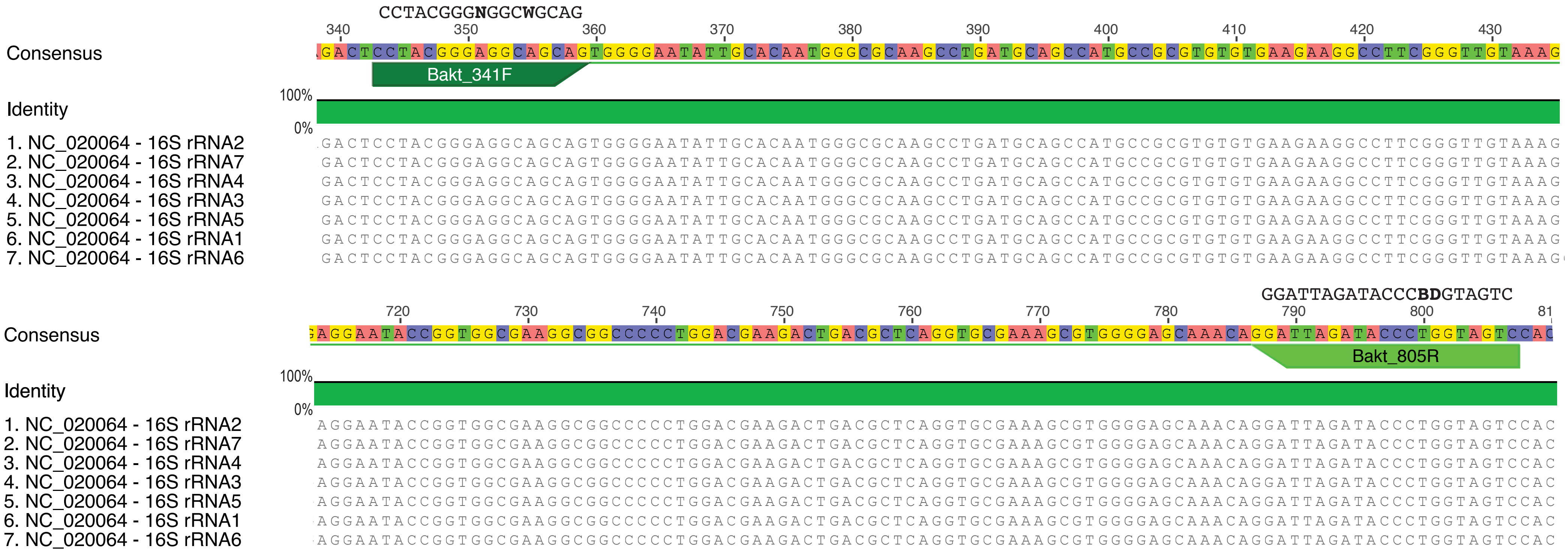
CCTACGGGNGGCWGCAG
340
350
360
370
380
390
400
410
420
430
Consensus
GACTCCTACGGGAGGCAGCAGTGGGGAATATTGCACAATGGGCGCAAGCCTGATGCAGCCATGCCGCGTGTGTGAAGAAGGCCTTCGGGTTGTAAAG
Bakt_341F
Identity
100%
0%
1. NC_020064 - 16S rRNA2
2. NC_020064 - 16S rRNA7
3. NC_020064 - 16S rRNA4
4. NC_020064 - 16S rRNA3
5. NC_020064 - 16S rRNA5
6. NC_020064 - 16S rRNA1
7. NC_020064 - 16S rRNA6
GGATTAGATACCCBDGTAGTC
720
730
740
750
760
770
780
790
800
81
Consensus
AGGAATACCGGTGGCGAAGGCGGCCCCCTGGACGAAGACTGACGCTCAGGTGCGAAAGCGTGGGGAGCAAACAGGATTAGATACCCTGGTAGTCCAC
Bakt_805R
Identity
100%
0%
1. NC_020064 - 16S rRNA2
2. NC_020064 - 16S rRNA7
3. NC_020064 - 16S rRNA4
4. NC_020064 - 16S rRNA3
5. NC_020064 - 16S rRNA5
6. NC_020064 - 16S rRNA1
7. NC_020064 - 16S rRNA6
